# Organizing work in local service implementation: an ethnographic study of nurses’ contributions and competencies in implementing a municipal acute ward

**DOI:** 10.1186/s12913-021-06869-4

**Published:** 2021-08-19

**Authors:** Helle Krone-Hjertstrøm, Bente Norbye, Birgit Abelsen, Aud Obstfelder

**Affiliations:** 1 Department of Health and Care Sciences, Faculty of Health Sciences, UiT The Arctic University of Norway, Tromsø, Norway; 2 Telemark Research Institute, Bø, Norway; 3 Department of Health and Care Sciences, Faculty of Health Sciences, UiT The Arctic University of Norway, Tromsø, Norway; 4 The Department of Community Medicine, The National Centre of Rural Medicine, UiT The Arctic University of Norway, Tromsø, Norway; 5 Department of Health Sciences in Gjøvik, Faculty of Medicine and Health Sciences, NTNU, Norwegian University of Science and Technology, Gjøvik, Norway

**Keywords:** Implementation, Primary health care research, Qualitative research, Nursing, Social organization of healthcare work, Articulation work, Organizing work, Nursing work

## Abstract

**Background:**

The increased prevalence of chronic diseases and an ageing population challenge healthcare delivery, particularly hospital-based care. To address this issue, health policy aims to decentralize healthcare by transferring responsibility and introducing new services in primary healthcare. In-depth knowledge of associated implementation processes is crucial for health care managers, policymakers, and the health care personnel involved. In this article, we apply an ethnographic approach in a study of nurses’ contributions to the implementation of a new inpatient service in an outpatient primary care emergency clinic and explore the competencies involved. The approach allowed us to explore the unexpressed yet significant effort, knowledge and competence of nurses that shaped the new service.

**Methods:**

The study combines observations (250 h) and several in situ interviews with healthcare personnel and individual in-depth interviews with nurses (*n* = 8) at the emergency clinic. In our analysis, we draw on a sociological perspective on healthcare work and organization that considers nursing a practice within the boundaries of clinical patient work, organizational structures, and managerial and professional requirements.

**Results:**

We describe the following three aspects of nurses’ contributions to the implementation of the new service: (1) anticipating worst-case scenarios and taking responsibility for preventing them, (2) contributing coherence in patient care by ensuring that new and established procedures are interconnected, and (3) engaging in “invisible work”. The nurses draw on their own experiences from their work as emergency nurses and knowledge of the local and regional contexts. They utilize their knowledge, competence, and organizing skills to influence the implementation process and ensure high-quality healthcare delivery in the extended service.

**Conclusions:**

Our study illustrates that nurses’ contributions are vital to coordinating and adjusting extended services. Organizing work, in addition to clinical work, is a crucial aspect of nursing work. It ‘glues’ the complex and varied components of the individual patient’s services into coherent and holistic care trajectories. It is this organizing competence that nurses utilize when coordinating and adjusting extended services. We believe that nurses’ organizing work is generally invaluable in implementing new services, although it has not been well emphasized in practice and research.

## Background

Norwegian health policy, like health policy in many other Western countries, aims to decentralize healthcare responsibility. This policy goal is due to demographic challenges involving an ageing population, an increase in chronic diseases, and insufficient coordination of health services. To address these challenges, governments initiate new services to achieve better, more effective health services and improve accessibility [1–3].

Several newly initiated services have originated from small-scale, locally developed service innovations considered successful in their local settings [4, 5]. Governments often “Scale-up and spread” [6–9] them as new services ready for implementation on a large scale nationwide without any further evidence or testing. They are often accompanied by financial and/or political measures, funding arrangements and an “ordering plan of translation from policy to practice” [1, 4, 10, 11]. However, several studies have shown that services that are successful in one context are not always successful in another [9, 12–15]. Many studies have pointed to the unpredictability of local translation, response, accommodation, and implementation [1, 14, 16, 17]. Health care professionals’ willingness to commit to local implementation processes is key to success [13, 15, 16, 18–20], as new and additional work is required [14, 21, 22]. To make a new service work professionals need to engage in work practices development, not just be willing to change [1, 8, 14–16, 18, 20].

This article draws on empirical material from an extensive qualitative study on how a new service, an inpatient municipal acute ward (MAW), was implemented at an outpatient emergency clinic. Our aim was to capture how the nurses contributed to the implementation process and the competencies that were involved in this work. We consider nursing work to be practised within the boundaries of clinical patient work, organizational structures, and managerial and professional requirements [23, 24].

### Theoretical perspective

Studies underpinned by *the theory of the social organization of healthcare work* describe how nurses perform *organizing work* and *direct patient work* simultaneously and continuously and highlight this combination as a crucial element of nursing work [22–26]. *Direct patient work* is the nurses’ clinical work, while *organizing work* involves care trajectories and organizing patient work. Both are different from administrative work, which is related to the management and leadership of the clinical units.

Regarding the theory *of the social organization of healthcare work*, one of its basic assumptions is that healthcare work is performed in complex organizations where responsibility for individual patients is distributed in time and space and across specialties. Healthcare work appears unpredictable and varied where quality and patient safety depend more on skilful information handling and resource allocation than on individual professionals’ clinical intelligence.

Consequently, there will always be some elements of healthcare work that need to be managed and negotiated in response to rapid changes in the patients’ conditions or ward resources. These continuous and prompt changes and the corresponding need for management are referred to as an *emergent organizing* [27, 28]. The concept *organizing work* captures how nurses collect and adapt information to ensure proper treatment and care for each individual patient [23, 25], as well as their ability to make sense of the complexity and other aspects of their nursing work.

*One key aspect of organizing work* is *articulation work. Articulation wor*k captures professionals’ practices in coordinating and aligning the varied elements related to individual patients’ care trajectory: “the work that makes the work, work” [25, 29, 30]. *It* connects the discontinuous and changing elements of professional practices and technologies, and requires the ability to respond to and handle emergent and unanticipated situations [22, 25].

Nurses perform much of this type of work and thus ensure coherence and quality in patient care. Nurses are present 24/7 and suited to perform this work. Through bedside care and the performance of tasks delegated by doctors and other professionals, they generate comprehensive knowledge and overviews of individual patients’ care needs, and how need, and care capacity changes.

In this study, we use these theoretical concepts as analytical lenses to observe, describe and understand how nurses work and contribute to the implementation of a MAW.

### Study background

In Norway, specialist care is a state responsibility, while municipalities are responsible for primary health care. In 2012, the Norwegian government introduced the Coordination Reform aiming at better and more effective healthcare for individual patients by transferring tasks and responsibilities from specialist to primary care. This shift led to more complex primary healthcare services that entail increased patient in-flow and new services for patients with more complex conditions and diagnoses [26, 31, 32].

One new service introduced by the Coordination Reform, aimed at patients who otherwise would be hospitalized, is a 24/7 inpatient MAW. This new service was inspired by locally developed rural health clinics [33] and community hospitals [34], described by the government as “successful in observing and treating patients near their homes, reducing hospitalization and achieving high scores in patient satisfaction surveys” [33, 35]. The government constructed a generic MAW model and required (since 2016) all municipalities to implement it as part of their primary health care service without any further testing or needs assessment.

The MAW guidelines provide a high degree of flexibility in how to organize and implement the MAW [36, 37]. This has resulted in considerable diversity in how MAWs are organized, and they vary in size, location and medical services provided [33]. The requirements and work descriptions for healthcare personnel working with MAWs are also relatively vaguely described [37–39].

Regardless of how a municipality organizes a MAW, a medical doctor has medical responsibility for the patients enrolled. The service requires a medical doctor for admission, for patient visits, and to be on call 24/7 [35, 37]. Nurses’ roles are described in terms of having a 24/7 presence and performing assessments immediately after admission [37]. Furthermore, the guidelines describe the need for *competent* healthcare professionals and describe general competencies, not directed to any particular profession, such as observation, decision-making, assessment, relational competence and the ability to handle acute situations [37]. Therefore, for the new MAW services to be put into practice, new routines, guidelines, checklists, protocols, capacity building and clarification of task divisions and healthcare professionals’ new roles to optimize their contribution must be developed and implemented. These are crucial elements in the implementation process. However, these aspects and the associated responsibilities are neither emphasized nor specified in the policy guidelines.

## Methods

This article draws on empirical material from an extensive qualitative study of how the generic MAW model was implemented locally, how nurses contributed to this process, and what competencies underlie their work. To explore this implementation process and aspects of nursing practice, we chose an ethnographical approach. As described by Côté-Boileau, Gaboury, Breton and Denis[40], the flexibility of the approach offered us the opportunity to capture how various institutional contexts, processes, and factors interacted and competed with each other through the implementation process and how this shaped the new service. We accessed a closeness to the dynamic relationships between these elements and processes enabling us to generate timely and knew knowledge about the importance of the nurses’ role in implementing the MAV. The need for knowledge of these aspects is highlighted in research [9, 28, 40, 41].

The nurses in this study practised in accordance with health policy guidelines, local organizational structures, professional discourses, and ethical conduct. These contextual factors and processes shape nurses’ experiences, expectations, work patterns, actions, and statements [42]. Health care professionals are not always aware of their actual contribution to local implementation work or do not portray certain professional skills or knowledge when describing their everyday work [23, 24, 43]. Using an ethnographic approach, we aimed to obtain deep insight into the organizational setting in which the MAW was established and knowledge of the nurses’ work when implementing the MAW as part of an existing municipal emergency clinic, which extends beyond the nurses’ own observations, interpretations, and articulations.

### Study context

The MAW is a generic model with considerable room for local adaptations. In the municipality where we conducted the fieldwork, the MAW was implemented as part of an existing outpatient intermunicipal primary care emergency clinic with a 24/7 service. The emergency clinic covered a large geographical area, with the nearest hospital two to three hours away. Occasionally, due to rough winter conditions, road access was limited.

Two nurses were responsible for the day-to-day management of the emergency clinic, and 14 nurses worked on a rotating schedule at the emergency clinic. Each shift was staffed with two nurses, doctors on call outside office hours, and one doctor responsible for MAW patients during office hours. The doctors on call worked at daytime as local GPs. The doctors are essential collaborators with nurses. However, the nurses represented continuity at the emergency clinic. Therefore, by design, the nurses’ perspective was prominent in our study.

Fieldwork was conducted early in the implementation process of the new service. The MAW expanded activities and responsibilities at the emergency clinic with an inpatient service, new and more advanced equipment, and a minimum of two nurses per shift [36]. The nurses were responsible for monitoring and caring for the patients admitted to the MAW in addition to operating the emergency clinic, including the communication centre, and follow-up at the outpatient clinic. The nurses also managed additional work involved in the implementation and switched between these new tasks and ordinary day-to-day duties as emergency care nurses. Based on competence and experience, the emergency nurses rotated between being responsible for operating the emergency clinic communication centre and performing triage over the phone, ordering transportation and receiving patients, while the other nurse assisted the doctor in the outpatient clinic, followed up, and monitored the MAW patients. In addition, the emergency nurses participated in “project groups”, each with different MAW implementation-related tasks, such as developing routines for ordering medicines and equipment and creating checklists, protocols, and routines.

### Data collection

The first author (HKH) conducted the fieldwork periodically from September 2014 to June 2015. Shadowing [7, 44] was used throughout the fieldwork because of the nature of the work and the patients admitted to the emergency clinic, who often had an immediate need for treatment and care. HKH accompanied the professionals and observed their everyday work within an organizational context, and by this performed “fieldwork on the move” [45]. This allowed her to access decision-making and sensemaking elements, which are often referred to as “invisible elements in health professional work”, and allowed close observation of the actual work carried out in the clinic [46]. HKH shadowed nurses and doctors on shifts; because of the nurses’ 24/7 presence, the nurses were more frequently shadowed. The process involved in situ interviews with nurses, nurse leaders, and doctors in the emergency clinic.

HKH observed who did *what*; *how* they performed different tasks, adjustments, and interactions with different materials such as guidelines, written texts, and people; and *where* their tasks were performed. The participants were presented HKH’s observations and interpretations in in-situ interviews and were given the opportunity to comment on them further during in-depth interviews [47, 48]. Continuously during the fieldwork, the nurses and doctors were asked to describe procedures, elaborate on their actions, and explain situations immediately after they occurred. This enhanced our understanding of their practices and daily work and corrected misinterpretations. For example, how frequently the nurses interacted with nurses and doctors at other local and regional service providers in performing daily care of their patients and in the implementation work. In addition, HKH and the co-authors AO and BN had frequent conversations during the fieldwork, and the different materials, observations and interpretations were discussed.

In the field notes, HKH noted keywords and quotations during the observations. HKH regularly took “writing breaks” to write complementary field notes with *thick descriptions* [49]. These field notes consisted of three parts: *first*, a general description of concrete observations or rendering of actual conversations; *second*, the author’s interpretations of the observations and immediate thoughts; and *third*, a more open approach with follow-up questions and possible links to written text.

In all, approximately 250 h of fieldwork was conducted, including a substantial number of field notes and numerous in situ interviews with nurses and doctors at the emergency clinic, in-depth interviews with two nurse leaders, and individual in-depth interviews with six nurses. The interview guides were influenced by our ethnographic approach and developed during the observation period. The different themes referred to the formal expectations of the MAW, local adjustments, and the informants’ experiences with the implementation process. The informants’ ordinary work at the emergency was also one of the themes. These guides served as a checklist during the interviews. Each interview lasted approximately 60 min, and each was performed during the informants’ work time. The interviews were audio-recorded and transcribed.

### Analysis

We used a stepwise inductive-deductive approach in our analysis. After the fieldwork was conducted, HKH read and marked field notes and transcribed text from the interviews. In this preliminary analysis, HKH searched for patterns, coherence, and activities that reflected the nurses’ work practice involved in implementing the MAW. HKH used the qualitative analysis software NVivo (QSR International Pty Ltd. Version 10) to sort and code the data. This process resulted in a coding framework with descriptive codes using observational passages and empirical terms and phrases representing the patterns identified. These codes and patterns were developed and discussed between all the co-authors. We then followed a more deductive approach, where the theoretical concepts guided the coding and further categorization of the coded data.

We especially paid attention to how the nurses described and reflected on the delivery and organization of the clinical patient work in the emergency clinic and what professional and structural consequences developed during the implementation of the MAW. Throughout the analysis process, we alternated between the empirical data and the theoretical concepts. As we gained insight and an overview of the data, we relied more on the theoretical concepts and were able to focus our analysis and develop explanations of our research aims. In this process, the initial coded text was divided into meaning units, condensed, and abstracted into codes (see Tables 1 and 2 for example) [50].

**Table 1:** Example of theme, subtheme, categories, subcategories, and codes from the analysis, interview material

Theme	The nurses’ organizing work					
**Sub-theme**	**Temporal articulation**		**Integrative articulation**		**Material articulation**	
**Category**	**Prioritize**	**Anticipate**	**Organize**	**Allocate**	**Adjust**	**Formalize**
**Sub-category**	**Sequence actions at the appropriate time and in the right order**	**Predictconflicting work pressure**	**Provide coherent trajectory elements**	**Negotiate and organize patient work (simultaneously)**	**Ensure (correct) materials are available to support their work.**	**Create a safety net**
**Codes (meaning unit)**	“Where does the MAW patient fit into all this?”	“How should we prioritize when things are busy”	“We need to have an overview and make sure every element is in place”	“How can we organize patient work when we can’t even agree on what a MAW is? Nevertheless, who else is going to do it?”	“Most of the things had to be adopted. We are an emergency clinic. We must make sure things are adapted to our needs here”	“We are completely dependent on having plans B, C and D as well as plan A”

**Table 2:** Example of meaning unit, condensed meaning unit, interpretation, subtheme and theme, observation material

Meaning unit	Condensed meaning unit	Interpretation of the underlying meaning	Sub-theme	Theme
Nurses discuss how they should prioritize when there are major medical actions, large patient numbers and the need to attend to the MAW patients at the same time.	The nurses identified and anticipated *conflicting work pressure* as a potential consequence of the MAW.	The nurses drew on their oversight of patient care, location, organizational knowledge and anticipated future consequences and worries about how necessary arrangements could be made.	**Temporal articulation**	**The nurses organizing work**
A nurse closes the MAW. She finds it irresponsible to have it open in rough weather conditions that can isolate the community. The doctor becomes frustrated and finds her decision irresponsible and re-opens the MAW.	Different perspectives exist regarding what a MAW is and should be in the clinic and the community in general.	The nurses argued for their perspectives when negotiating for what they considered *sound medical care*, and at the same time made new arrangements to ensure the MAW was interconnected with the overall service.	**Integrative articulation**	
A critically ill patient is admitted. A nurse explains how he/she did not see patients like that before, e.g., “My instinct as an emergency nurse is to send him to the hospital. Now I am responsible for monitoring and taking care of him while operating the emergency call centre”.	The MAW requires new care arrangements and skills to provide this type of care and treatment.	MAW forced the nurses to see both the clinic and their work in a new and different context and perspective. To handle the new situation, the nurses wrote up new- and altered procedures, routines, etc. Through this, they *created a safety net* and concretized the MAW.	**Material articulation**	

Early in the analysis process, we identified the relatively invisible but substantial element in the nurses’ everyday work practice, *the nurses organizing work*. Furthermore, we identified aspects of the *nurses organizing work* as *articulation work* and how these aspects of nursing work were relevant when analysing and understanding the nurses’ contribution to the implementation process. We realized how the nurses had the ability to make sense of the complexity in the healthcare service and the chronological aspect of patient work and utilized this competence in the implementation process.

Tables 1 and 2 illustrate how the theme of the *nurses organizing work* was categorized into subthemes of different types of *articulation work.* The codes and meaning units are abstracts of empirical phrases, quotes and observations from the fieldwork, representative of the patterns. In Table 1, the categories refer to the empirical terms we used in the coding process, and the subcategories are our analytical description of them. The second column in Table 2 is a condensed description of an observation, and the third column illustrates an analytical interpretation of the underlying meaning from the coding process. The codes and meaning units were analysed and discussed between all the co-authors and further developed by writing the article manuscript. The result of this dynamic analytical process is presented below (see Table 3 for key findings).

**Table 3:** Overview: key findings

The nurses’ organizational work		
**Temporal articulation**	**Integrative articulation**	**Material articulation**
The nurses drew on knowledge and overview of individual patients need for care and the capacity of the care environment to accommodate these and anticipated potential consequences of *conflicting work pressure.*	The nurses argued the importance of matching the potential patient’s need with the capacity of the care environment to adequately respond to them and demonstrated a holistic understanding when negotiating local adjustment of the MAW.	The nurses identified how the MAW required greater overview over care arrangements and contributed to formalizing these aspects in the clinics’ framework through writing up new and revised procedures.
The nurses worked to determine how to sequence the action and prioritize the patients in the new, extended service.	The nurses contributed to ensuring that the MAW was interconnected and facilitating coherence in the new extended service.	The nurses undertook great responsibility for establishing and securing access to material to support the work at the clinic and creating a *safety net.*

### Ethical considerations

 This study was carried out in accordance with the Declaration of Helsinki (World Medical Association). It was approved by the Norwegian Centre for Research Data (reference: 3889) and the Regional Committees for Medical and Health Research (reference: 2014/1658). Written information about the study, including the participants’ legal rights regarding participation and confidentiality, was provided. The participants were assured that participation in the study was voluntary and that they were free to withdraw from the study at any time. All participants provided their informed consent to participate. However, the data sets generated and analysed during the current study are not publicly available due to the lack of obtained consent among the participants to share raw data but are available in Norwegian from the corresponding author upon reasonable request. Anonymity and confidentiality were assured according to the standard procedures, and the participants’ names and other directly identifying information were anonymized in the written text.

## Results

The implementation of the MAW involved a reordering of established work patterns, collaborative practices, and competencies. This reordering does not occur automatically or in a vacuum; rather, it occurs as a result of ongoing negotiations, adaptations, translations and adjustments of activities and responsibilities. In the results section, we describe different aspects of the nurses’ *organizing work* that contributed to interconnecting the MAW with the overall workflow at the emergency clinic. The excerpts presented under the results sections are therefore an expression of the nurses’ reflections on their present daily work life and situation and past and potential future of the service. In our analysis, we categorized aspects of the nurses’ *organizing work* as three types of articulation work: *temporal, integrative*, and *material articulation*. These three types of work, which are heavily depended on oral and written communication between the nurses and other colleagues, are not mutually exclusive and are performed separately, simultaneously and continuously.

### Temporal articulation

The first aspect of *the nurses organizing work* we are highlighting is the nurses’ temporal articulation, i.e., how the nurses coordinated their actions in the emergency room in *time* and how they appropriately identified, prioritized, and delegated tasks and activities. Temporal articulation requires familiarity with the organization and practices of the emergency clinic, including understanding of both their own and their colleagues’ roles and what is expected of them at all times and in different situations. Our analysis shows that the implementation of the MAW disturbed the established organization and practices of the emergency clinic, entailing new needs, expectations and practices.

The nurses had influence over how the MAW should be integrated into the other work in the emergency clinic and how to organize the work to ensure care for both inpatients and outpatients and the smooth running of the emergency call centre. The nurses worked to determine how to sequence their actions, act at the right moment, and prioritize patients with this new service. The quotation below shows how a nurse described her work process in receiving emergency clinic patients. She expressed the need for new procedures to care for MAW patients and her uncertainty regarding how MAW patients fit into the existing “care pathway” of the clinic. She explained that she starts preparing for the eventual arrival of a patient from the moment that she receives an emergency call:

*Excerpt 1*

*“(…) I already have an idea of whether I need to consult with someone or tell a doctor directly. What’s the degree of urgency? Do I need to make an appointment, or does the patient have to be prioritized and go straight to the doctor? Should I call patients living a long way away and ask them to wait before they come here? All this rushes through our heads when we talk to the patient on the phone and when we’ve hung up. Although it may seem chaotic when I’m talking about it now, we can handle it. We can. Where the MAW patient fits into all this – and everything that needs to be in place here… well, no, it… what are their needs? How should we prioritize things, all that (…)?” (Interview, Nurse)*

This quotation illustrates how the nurse matches the patient’s potential care needs and the care arrangement in the emergency clinic. The nurse expressed concern about how to care for a MAW patient within the established practices and procedures of the clinic. This demonstrates how MAW patients necessitate new procedures, new care pathways and a change in emergency clinic capacity and culture. Nurses have an established procedure for caring for outpatients and for operating the emergency clinic. They say they know their role and what is expected of them in this work. They deal with unpredicted events on a daily basis. In their work, they experience and solve problems rapidly, and this work can sometimes be very demanding, requiring full concentration. Nurses are also familiar with the available resources and materials in the clinic and have knowledge of local factors. The emergency clinic is located in an area where the weather can be harsh, roads may be closed at short notice, helicopters can have difficulty landing and the nearest hospital is a two-hour drive away. Nurses are used to addressing these issues in the traditional framework of emergency clinic work. HKH observed that the nurses imagined (and feared) possible *conflicting work pressures*, where they had to attend to and monitor critically ill inpatients in the periodically hectic and chaotic outpatient situation of the emergency clinic. The analysis revealed that the nurses were worried about this issue, especially in discussions about the location of the MAW and difficulties operating an outpatient emergency clinic with an inpatient service:

*Excerpt 2*

*(…) What happens when there are major medical actions and everything’s boiling over? Because it’s sometimes like that here in the emergency clinic. We’re a long way from hospitals and we’re looking after patients who other clinics would have sent straight to hospital. I can give you a specific example of that. Imagine there’s been a major accident, or there’s a big medical action, while at the same time patients are flocking in here. What happens to the MAW patient then? (…) How should we make priorities in all that? How should we solve it in a professional way? (…) I truly wonder whether MAW should be in the emergency clinic. We don’t operate with beds. Here patients are in and out all the time. In addition, who’s left with the responsibility in practice? An MAW doctor who comes in briefly in the daytime and barely sees the patient? Emergency clinic doctors should not be responsible unless it is acute. So I’ll tell you who’s responsible; it’s us nurses. It does not seem very responsible when we have to be in charge of the emergency number and the clinic at the same time to be honest. Although I understand that we have to have it (…). (Excerpt from field notes, two nurses)*

In this quotation, the nurse describes external factors that are difficult to control, but they appeared to be important factors in the context of the nurses’ work. At this time, the MAW was relatively new, and the nurses’ uncertainty, as we interpret it, may have been related to two underlying factors. *First*, regarding the clinical aspect, the MAW involves a new type of patient who requires a different type of attention and may have different needs than the typical emergency clinic patient, and the nurse is unsure what this will require in terms of her attention and clinical skills. *Second*, the nurse is worried about whether the clinic as an organization has the capacity to attend to the MAW patients in all situations. Our data showed that the nurses anticipated the potential consequences of the MAW, such as patients in need of observation, care and treatment that the emergency clinic had not previously provided, at an early stage.

### Integrative articulation

The second aspect of *the nurses organizing work* we wish to highlight is nurses’ *integrative articulation*. This term refers to their “logistical” work to ensure that all elements of a care pathway are connected, ensuring that care arrangements and decision-making are coherent. For the MAW pathway, inpatient admission procedures had to be established, with clear responsibilities for the care of MAW patients and the provision of necessary materials.

In the MAW guidelines, there was a high degree of flexibility in how the service should be organized; however, there were a few requirements regarding the type of patients to be allotted beds. Nevertheless, the nurses found it unclear how this procedure would work in practice in the emergency clinic, how it would be incorporated into other practices, and what the MAW patient (the organization around the patient) would require. There was a great need to make local adjustments. Our data showed that these local adaptations were challenging to develop and implement, partly because of an unclear distribution of roles and disagreements regarding the MAW itself. There was disagreement among the different doctors and nurses regarding what they considered the responsible way to run the MAW in the clinic and for the community. This disagreement was expressed in discussions in the emergency clinic:

*Excerpt 3*

*A nurse starts her report by stating that she has decided to close the MAW for the weekend. “It’s the weekend and very strong gales are forecast for the next 24 hours (…). We risk not getting patients to the hospital if they become acutely ill when they are admitted here. We simply cannot risk having no ambulances available, and the helicopter will definitely not be able to land in those conditions”. Later, the doctor on duty arrives and is very frustrated with the decision: “If we close the MAW now it’s a real crisis. If we cannot get patients sent to the hospital, we need the MAW more than ever. If there’s no emergency transport, these two beds are all we have to offer”. (Excerpt from field notes, two nurses and a doctor)*

As the quotation illustrates, there were different perspectives on what the MAW should offer in the clinic and in the community in general. Although doctors have medical responsibility for patients at all times, the nurses found that they were ultimately responsible for observing and caring for the patients 24/7. Several of the nurses said that nurses in an emergency clinic needed to acquire further knowledge and skills to ensure that high-quality care was provided. Our impression was that the nurses had a perspective, including work in the clinic with the local infrastructure and the weather conditions, that could prevent transportation to the hospital if needed. We observed that the nurses were negotiating among themselves and with doctors about the admission of patients to the MAW. In the break room, they negotiated “sound medical care”, which we interpreted as the basis for treating patients.

*Excerpt 4*

*“The requirements are relatively clear as to who should be in the MAW beds, which has meant that only a few patients actually qualify – or are assessed as qualifying (…). We nurses and the doctor don’t always agree, but it’s always the doctor who has the last word, of course. However, sometimes we need a vacant observation bed. Is the treatment we’re giving the patient effective, or do we need to transfer the patient to the hospital? That’s the simple choice. Then, the MAW is an observation for a few hours. We have to be flexible, too (…). We just do it. I’d just like to clarify something. We can get as annoyed as we want at the management, politicians or the municipal health service. However, ultimately, when we’re here on the job, it’s for the patients; the patients are at the centre, they’re our focus. They’re the people we have to take care of. In addition, I promise you, patients here are being taken care of. No matter how much effort we have to put in, adapting everything. That’s just the way it is. Because who else is going to do it?”* (*Interview quotation, nurse*)

The disagreements observed were largely related to the challenges and opportunities related to the MAW being one of several services that, in combination, are intended to provide services to the inhabitants, and the MAW, according to the national health authorities, should be operated as an *instead of hospital* service [35]. This entails a service that provides healthcare to patients that is equal to or better than that provided by hospitals. The work involved in implementing the MAW was clearly important to the nurses, along with the perception that the service should be well integrated into the emergency clinic and into overall municipal health care provision. The complex work in ensuring that decisions and procedures are interconnected and dealing with the different perspectives of the MAW was demanding, and there was much disagreement about how the MAW should be used both in the emergency clinic and in the other healthcare services.

### Material articulation

The third aspect of *the nurses organizing work* concerns *material articulation*, i.e., “work that ensures the materials are available to support the work” [23]. In the previous sections, we indicated how the nurses worked to identify, adapt, prioritize and allocate various tasks involved in the organization of patient care, sequencing them in a sensible manner and linking the various actors both within and outside the MAW. Supporting this work, *materials* refers to technology, expertise, resources, text, and proper equipment. The nurses worked actively to ensure that the right equipment and resources were in place and ready for use as needed.

When the MAW was introduced, the guidelines did not specify the resources and materials that would be needed to run the MAWs as part of the everyday practice of the emergency clinic. As we have shown, the MAW necessitated new practices in the emergency service and, thus, also new procedures that were suitably adapted. We observed that the nurses worked to ensure that there were procedures to address the new situation and organized working groups for this purpose.

MAW patients require different care needs and attention from nurses than typical emergency unit outpatients. In addition, the organization of the MAW patient work required a broader collaboration with services that previously had not been involved to the same degree. The nurses had to discharge and transfer patients to other primary healthservices, such as nursing homes and home nursing care. Accordingly, the nurses had to make transfer arrangements and ensure that the patient discharge summaries and medical records were completed.

Working with MAW patients in the clinic, the nurses also had to have a new understanding of both their own and their colleagues’ role and activities and what was expected of them at all times, such as new procedures for monitoring critically ill patients 24/7. Traditional patients at the emergency clinic did not require this type of procedure or clinical skills. This also required a greater overview of the care arrangements and possibility for immediately being sent to the hospital via helicopter or ambulance, as well as an assessment of how soon the doctor could be there or what the weather conditions were. The nurses expressed a need to include these new arrangements in the framework of the emergency clinic, partly by drawing up new and revised procedures.

*Excerpt 5*

*“(…) Distribution of responsibilities. Who orders things; we even had to make a system to decide who would make a system. Because in all of this, things have to fit in with our emergency clinic – the standard item that’s included(…) no, it doesn’t fit in here. So we’ve made new medicine lists, treatment plans, ordering procedures, descriptions of duties, procedures… yes. Most of the things had to be adapted, in fact (…). We’re an emergency clinic. If we’re going to be innovative, we must make sure things are adapted to our needs here (…). We must have procedures for who does what, are you on MAW or clinic today, who’s in charge of the emergency call centre. In addition, things happen quickly in an emergency clinic. Suddenly, it’s all go here; it may be medical actions or accidents. We’re completely dependent on having plans B, C and D as well.” (Interview quotation, nurse)*

As the quotation illustrates, the nurses took responsibility for establishing the necessary procedures, which ranged from ordering supplies and new equipment to record keeping for the MAW patients. They identified needs based on their experience and nursing expertise and worked hard to ensure that the necessary materials were in place for the MAW patients.

The introduction of MAW beds meant that the nurses had to observe and care for critically ill patients for longer than they usually did in the emergency clinic. Our data show that several of the clinic staff described challenges because the clinic was not organized to provide this type of care and treatment. The nurses expressed a need for competence that would enable them to perform their job and provide “sound medical care” and the resources and materials to support their work.

*Excerpt 6*

*(…) I’ve put in an IV catheter, given intravenous medication, handled a syringe driver and used a CPAP machine. We often have patients with COPD, for example. Then, it’s important to have oxygen in the cupboard. It is often chronically ill and/or geriatric patients who are in here for a short time; these are typical MAW patients. We didn’t see patients like that before. We met them in a more critical phase where we sent them off for hospital treatment. Now they’re here and will stay here with us. We’re to observe them, treat them, look after them over time. Is the patient deteriorating? Is the patient responding to treatment? Should a doctor take a look now? Can we send them home? What care will they get after that? They also often have lists of medicines running to a whole page from the nursing home or home care. This is quite a change. (…) Suddenly, we were without oxygen here and had to pass patients on. Because we didn’t have a routine for ordering it as frequently as we actually need it now. (…) We have to have the necessary medicines on those long lists and equipment to care for patients over time, good cooperation with the other health care facilities that maybe also know the patient. (…) So it’s not necessarily a whole new patient group, but the way we work and what this requires of us nurses and the clinic is different.*

*(Excerpt from field notes, nurse)*

The nurses worked to gain expertise in observing and caring for the MAW patients by providing what they considered “sound medical care”. Our data show that the MAW did not always bring a new patient group to the emergency clinic but involved treatment that was previously the responsibility of the hospital. The nurses also had to care for these patients over time. At an early stage, this was described as a *learning by doing process*. Our data showed that the nurses worked hard to acquire knowledge and skills to care for the MAW patients while at the same time establishing routines and procedures and taking responsibility for the clinic as an institution that could receive the patients and provide them with high-quality health care.

## Discussion

The MAW is an initiative that represents current changes in municipal healthcare [36]. New, complex services are introduced in municipal healthcare services without further evidence or testing and with relatively vague guidelines for operationalization [1, 6]. Interventions that are considered successful in one context are implemented across systems within a “Scale-up and spread” framework [9]. Several studies have shown how additional work is required when new services are being implemented in an already complex health care delivery setting [14, 20, 34]. Furthermore, researchers have pointed to the importance of understanding how the local context shapes implementation processes [9, 41] and including local contextual elements when understanding the variation in success in implementing new services from one context to another [9, 28]. Nevertheless, as previously described, there has been little research on identifying the *actual work* and the contextual factors involved in these local implementation processes or the competencies required. Our methodological approach has allowed us to generate contextualized knowledge of relationships, materials, competencies, cultures, and expectations of good care in and across local and regional service providers influencing local implementation work and the nurses’ contribution to this process.

### Anticipating worst-case scenarios and taking responsibility

The nurses drew on clinical and organizational knowledge and contextual resources and factors in their implementation work. In our analysis, we became aware of the importance of contextual factors and how central they were in addition to clinical and organizational knowledge, when anticipating different worst-case scenarios and how they, through creating an extended “safety net”, took responsibility for preventing them.

The emergency clinic is an outpatient service that delivers acute, episodic care to inhabitants in thinly populated municipalities in northern Norway, located two to three hours from the nearest hospital and occasionally subject to rough winter conditions. Various organizational frameworks, such as procedures, materials, and the established division of labour, supported the complex and, at times, challenging work of the emergency clinic. In contrast, this new inpatient service they became responsible for was not accompanied by a “safety net” of concrete procedures, resources, and defined resources and tools.

The nurses contributed to actualizing the MAW’s guidelines by developing and adjusting this ‘safety net’ in accordance with the extended responsibilities. Our analysis illustrates that in this work, they drew on (local) knowledge of the municipalities, including knowledge of other related services, their own clinical experience as nurses at the emergency clinic and knowledge of the patient population. Furthermore, their knowledge included geographical contextual factors, such as weather conditions and distances, in addition to professional discourses, organizational structures, local and national health policy, and other relevant contextual factors [42, 51].

Our analysis illuminates how the nurses contributed to the implementation process by bringing together these different elements they considered relevant to the new extended service. To ensure that the patients were cared for in what they considered a “sound medical way”, they shaped specific and dynamic configurations of contextual, material and human elements. These care arrangements represented a new and alternative “safety net”, which provided them with confidence that they could deliver high-quality care at the new extended service.

Drawing on *the theory of the social organization of healthcare wor*k [23, 24], we understand these ordering aspects of nurses’ implementation effort, such as articulation work, a particular aspect of nurses’ organization work [23]. As described earlier, nurses’ work includes both clinical and organizing aspects. Over the years, society has changed, and institutions and work tasks have naturally changed with them. As Allen [23] argues, nurses’ work is practised within the boundaries of clinical patient work, organizational structures, and managerial and professional requirements [23, 24], and part of taking care of the patients is also paying attention to the environment at the institution and the contextual factors. The nurses utilized their knowledge in the overall implementation process, from identifying and anticipating potential risks, ensuring that the MAW service was coherent through allocating tasks and negotiating new practices to adjust the MAW to the outpatient clinic, and formalizing the knowledge through the development of procedures and guidelines. We argue that the nurses *organizing work*, particularly the aspects of *articulation work*, enabled the nurses to handle this new implementation work in a partially intuitive manner that is effective and agile [18].

### Contributing coherence in patient care

Nurses recognize that external factors that are difficult to control can still affect the treatment and care of patients [52]. The nurses identified at an early stage that taking care of and monitoring critically ill patients in a MAW required a greater overview of the care arrangements that included contextual factors, such as weather conditions and the possibility of immediately transfering patients to the hospital via helicopter or ambulance, in addition to available doctors and competence. The nurses continuously negotiated for what they considered “sound medical care”, which, to our understanding, included aligning complex and varied components of the individual patients’ services into coherent and holistic care trajectories.

In healthcare, the power relationship between doctors and nurses is a key factor. Traditionally, nurses have been dependent on doctors’ support and collaboration [24, 53, 54]. In our study, the work relationships between doctors and nurses were not well established. There was a high degree of doctor turnover, and the doctors on duty varied throughout the week; therefore, the nurses represented personnel continuity at the clinic [24, 55]. Nevertheless, based on our findings, the doctors were considered essential collaborators in interpreting and operationalizing the MAW and were an essential part of the clinic’s professional framework [52].

As our results illustrate, doctors and nurses often negotiated admissions and “sound medical care” in the operationalization of the MAW. Both the nurses and doctors operated within the same context. Nevertheless, they did not always share the same interpretation of the MAW, which patients should be admitted and what was considered an adequate MAW service. The nurses demonstrated a holistic understanding of patients’ needs and capacities at the emergency clinic when negotiating local adjustments of the MAW. Their 24/7 presence at the emergency clinic generated comprehensive knowledge of patients’ needs for care and the capacity of the care environments to adequately respond to them.

Health care work is complex, and there will always be elements of the work that are difficult to control, which necessitates ongoing management and negotiation and what Allen refers to as *emergent organizing* [27, 28]. In their negotiations, the nurses argued that there will always be some elements in patient care that need to be managed and adequately addressed in immediate response to a patient’s condition and available resources. Therefore, they negotiated the importance of both ensuring that the MAW was well integrated and cohesive with the overall workflow at the emergency department and extending the framework to be able to make arrangements that correspond with the potentially critically ill MAW patients’ needs.

To be able to handle their work and pressure under changing circumstances, the nurses were more conscious and relied more on a “safety net” as a response to prevent “worst-case scenarios” than the doctors. The doctors, who often had the ultimate medical responsibility, had the final word, thereby contributing to pushing the service forward [1].

Our analysis illustrates how the nurses continuously brought together different elements of patient care to ensure that the patients were cared for through what they considered “sound medical care” and followed general MAW guidelines. Through the various types of articulation work, the nurses developed and shaped specific and dynamic care arrangements, ensuring good patient care and well-being, safety for the nurses and at the same time support the doctors’ work. Through this, they contributed to the “gluing” of the complex and varied components of individual patients’ services into coherent holistic care trajectories and to the quality assurance of the services.

### Invisible work

*Nurses organizing work* is a relatively invisible element in everyday nursing practice [18, 23]. Several studies have described nurses as being expected to perform and take responsibility for “unspecified tasks” [23, 24, 53]. For example, they identify, assess, and perform other crucial tasks necessary for providing care that are not specified or formally designated responsibilities [23, 24]. These unspecified tasks are often “invisible” and taken for granted, despite being crucial for collaborative relationships and work [43, 56, 57]. Our analysis illuminates how the nurses in our study took responsibility for “unspecified” implementation tasks as part of their *organizing work* and made them “specified” by establishing dynamic care arrangements.

According to both the MAW guidelines [37] and the Health Personnel Act [58], doctors have the ultimate medical responsibility. However, the responsibility for implementing and operationalizing new services is not specified, even though these are crucial and essential elements of health care delivery [17, 59]. The nurses in our study took on this responsibility and managed this new and additional work, for example, through developing “safety nets” and linking formal and informal resources to organizational, contextual and professional structures [54, 60]. Our analysis illustrates that the nurses’ organizing work enabled the nurses to handle this new implementation work in a partially intuitive manner. The nurses drew on their competence when developing and adjusting the ‘safety net’ in accordance with the extended responsibilities by developing and formalizing relatively vague guidelines and thereby contributing to implementing the new service at the emergency clinic.

In accordance with several studies on the organization of work by health professionals [23, 24, 43], the nurses in our study did not portray certain professional skills or knowledge when describing their work and were not always aware of their actual contribution in implementing the MAW. Rather, when asked, the different contributions and tasks were referred to as “*just a part of our work as nurses*” or “*off course we do this, who else is going to do it*?”. Our methodological approach enabled us to explore these aspects of their work. When the nurses reflected upon and discussed the MAW as an inpatient service in an outpatient emergency clinic, the nurses established work patterns, collaborative practices, and competencies that became visible. This allowed us to describe, systematize, and analyse elements of nursing work, competence, and knowledge they used to influence the implementation process. During the research process we revealed how a contemporary rural service provider operated when responding to a national governmental initiative. Our approach offered an entry point to produce timely and contextualized knowledge of how the new emergency service was shaped which we believe can inform stakeholders in health and care service innovation and organisational changes.

Our analysis illustrates that nurses contribute to the vital coordination and adjustment of the new service [18, 22, 61]. According to Allen and May [28], *“high-quality healthcare depends not on individual brilliance but on ensuring that all the necessary elements (material, knowledge, people) to meet patient needs are aligned in social time and space*”. Our findings illustrate how nurses performed this varied work in developing and implementing the MAW and developing new professional and organizational practices. Therefore, they also contributed to developing high-quality healthcare.

We believe that nurses’ relatively invisible organizing work is generally invaluable in implementing new services and is a substantial contribution in ensuring that services are considered comprehensive for individual patients, although this point has not been well emphasized or acknowledged in practice and research. Several studies have pointed to the importance of implementation processes and operationalization when introducing new services [9, 11, 12, 15, 62]. Our research describes the *actual work* of nurses in local implementation work, sheds light on their contributions and makes visible various elements of nursing work.

## Conclusions

The success of implementing new services in healthcare delivery has been described in several studies as depending on the organizational culture, collaboration and willingness of the involved healthcare workers and the ability to overcome a “reluctance to give up old habits” [63–65]. In accordance with Lydahl [18], we also argue that making new services work in practice depends on highly developed skills and competence, not just willingness to change. Our findings illustrate nurses’ contributions through engagement, activities, and initiatives that are essential for coordinating and adjusting extended services. Nurses’ organizing work, in addition to their clinical work, is a crucial aspect of nursing work. However, nurses’ organizing work is invaluable in implementing new services and should not be part of the invisible work taken on as individual responsibility. Future implementation efforts should take this work as actual organizational work into account, and it should be acknowledged as an important and invaluable contribution.

### Implications

The requirements for quality and competence set by the reform are more challenging for rural municipalities to meet [36, 66]. We argue that the tendency of decentralization of tasks and responsibility, from specialist to primary healthcare, could have more impact in rural areas far from hospitals and contribute to nurses’ substantial involvement in the implementation processes.

### Limitations

We have no data on the involvement of municipal leaders and politicians or doctors engaged in this new service. Nevertheless, we acknowledge that our focus in this study was on nursing work. An essential aspect of the context is other professionals. Understanding the implementation processes’ complexity cannot solely be achieved by analysing parts, and one profession works independently. We consider it both a strength and potential weakness that we in this article focus on the nurse’s work. We did not interview doctors specifically, municipal leaders, politicians, or other healthservices on the subject of implementing and developing the MAW, which could be of interest for further research.

## Data Availability

All participants provided their informed consent to participate. However, the data sets generated and analysed during the current study are not publicly available due to the lack of obtained consent among the participants to share raw data but are available in Norwegian from the corresponding author upon reasonable request. Anonymity and confidentiality were assured according to the standard procedures, and the participants’ names and other directly identifying information were anonymized in the written text.

## Abbreviations

MAW: Municipal acute ward
GP: General practitioner
COPD: Chronic obstructive pulmonary disease
